# Cardiac Troponins as Biomarkers of Cardiac Myocytes Damage in Case of Arterial Hypertension: From Pathological Mechanisms to Predictive Significance

**DOI:** 10.3390/life12091448

**Published:** 2022-09-19

**Authors:** Aleksey Michailovich Chaulin

**Affiliations:** 1 Department of Cardiology and Cardiovascular Surgery, Samara State Medical University, 443099 Samara, Russia; alekseymichailovich22976@gmail.com or a.m.chaulin@samsmu.ru; Tel.: +7-(927)-770-25-87; 2 Department of Histology and Embryology, Samara State Medical University, 443099 Samara, Russia

**Keywords:** pathophysiology, myocardial injury, cardiomyocytes, apoptosis, cardiac troponins, predictive significance

## Abstract

Background. Many pathological conditions of both cardiovascular and non-cardiac origin in the course of their development cause damage to contractile cardiac muscle cells—cardiac myocytes (CMCs). One of the most sensitive and specific criteria for detecting CMCs are cardiac troponins (CTs), which are regulatory protein molecules that are released into the blood serum from CMCs upon their death or damage. New (high-sensitive) methods for detecting CTs allow the detection of minor CMCs damages at the earliest stages of cardiovascular diseases and can therefore change the understanding of disease development mechanisms and open up new diagnostic possibilities. One of the most common and dangerous early diseases of the cardiovascular system is arterial hypertension. The purpose of this paper is to summarize the pathophysiological mechanisms underlying CMCs damage and CTs release into the bloodstream in the case of arterial hypertension and to state the clinical significance of increased CTs levels in patients with arterial hypertension. Materials and methods. This is a descriptive review, which was prepared using the following databases: Embase, Pubmed/Medline and Web of Science. The following key words were used in the literature search: “myocardial injury” and “arterial hypertension” in combination with the terms “cardiac troponins” and “mechanisms of increase”. Conclusions. According to a literature analysis, CMCs damage and CTs release in the case of arterial hypertension occur according to the following pathophysiological mechanisms: myocardial hypertrophy, CMCs apoptosis, damage to the CMC cell membrane and increase in its permeability for CTs molecules, as well as changes in the glomerular filtration rate. Most often, increased CTs serum levels in case of arterial hypertension indicate an unfavorable prognosis. Data on the CTs predictive significance in case of arterial hypertension open the prospects for the use of these biomarkers in the choice of patient management plans.

## 1. Basics of Biochemistry, Physiology and Diagnostic Contribution of Cardiac Troponins

Cardiovascular diseases are one of the most common causes of mortality and disability of the world’s population; therefore, the search and discovery of new diagnostic capabilities is a priority in current research [1,2,3]. The contribution of cardiac troponins (CTs) in the modern diagnostics of cardiovascular diseases cannot be overestimated. These laboratory biomarkers are considered the most sensitive and specific indicators of myocardial injury, which allows them to be considered as the “gold standard” for diagnosing acute coronary heart disease (CHD)—myocardial infarction (MI) [4,5,6,7,8]. CTs are protein molecules that, together with another protein called tropomyosin, form the troponin-tropomyosin system, which is an important component of thin (also called actin) filaments [9,10,11]. Among the proteins that are part of thin filaments, only cardiac troponin T (CT-T) and cardiac troponin I (CT-I) have a specific structure characteristic of the main cells of the heart muscular membrane—contractile cardiac myocytes (CMCs). All other thin filament proteins, including actin, tropomyosin and troponin C, have the same structure. CTs are significant contributors due to participating in the regulation of the contraction-relaxation of operating CMCs. The name of cardiac troponins is in accordance with their function. Troponin C binds to calcium ions, which enter the cytoplasm mainly from the sarcoplasmic reticulum, in which calcium channels open when a nerve impulse is transmitted. CT-T provides the attachment of the troponin–tropomyosin system to thin filaments and, after troponin C binding to calcium ions, participates in the conformational (spatial) movements of the troponin–tropomyosin system for the subsequent opening of myosin heads binding areas on actin. The interaction of the last two proteins with the formation of transverse actomyosin bridges underlies the contraction of striated muscles. CT-I, on the contrary, is active during muscle relaxation and blocks the formation of actomyosin bridges. Mutations in the genes encoding CTs lead to the pronounced disorders of CMCs contraction–relaxation (hereditary cardiomyopathies), which are clinically manifested by a group of symptoms of heart failure (HF) (shortness of breath, general weakness, defatigation, edema, etc.) [12,13,14,15].

Proteins CT-T and CT-I are released from the cell into the blood during the ischemic necrosis of CMCs, which can be used in the diagnosis of MI [4,5,6,7,8]. The use of CTs for the diagnosis of MI is regulated in a number of modern recommendations of the European, American and Russian Cardiological Societies [8,16,17,18].

**Figure 1:**
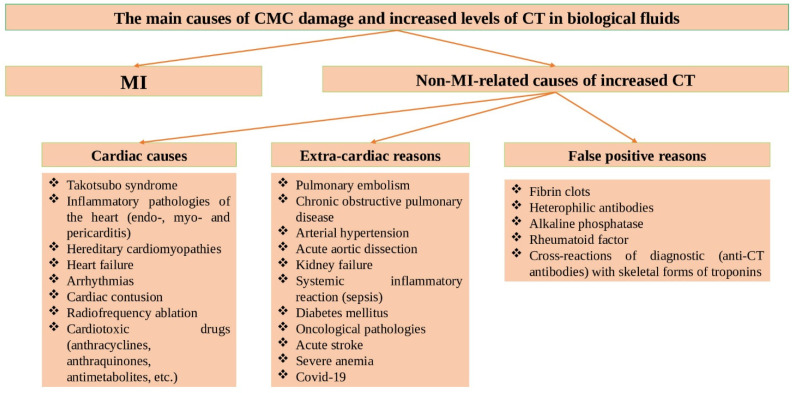
Main reasons of CMCs damage and increased CTs levels in body fluids. List of abbreviations. CT—cardiac troponins; MI—myocardial infarction, CMC—cardiomyocytes.

Although currently the main area of CTs application in routine practices is the diagnosis of MI, the diagnostic significance of these biomarkers is far beyond this acute cardiovascular disease. This is confirmed by a number of clinical and experimental studies that reported positive levels of CTs in the blood of patients who had non-cardiac diseases (renal failure, stroke, chronic obstructive pulmonary disease, diabetes mellitus (DM), those who took cardiotoxic drugs to treat underlying cancer, and other causes) [19,20,21,22,23,24,25,26,27], and non-ischemic cardiovascular diseases (cardiac tachyarrhythmias, cardiomyopathy, myocarditis, HF, and a number of other causes) [28,29,30,31]. Some researchers report diagnostically significant increases in CTs levels during physiological processes (for example, during physical activity [29,31,32], the effect of stress on the human body [33] and age- and gender-related features of CTs concentration [34,35], as well as the effect of daily biorhythms to CTs levels) [36,37,38,39]. In general, these papers indicate the presence of many other mechanisms (not caused by ischemic necrosis) of CTs release from CMCs. Therefore, CTs may indicate the presence of MI only if patients have symptoms of myocardial ischemia (chest pain typical for MI, ischemic changes detected using electrocardiography and echocardiography). Many papers report a very high number of non-MI-related cases of increased CTs serum level. For example, according to a large retrospective study by Lindner et al., almost 90% of cases of increased CT-T were caused not by MI but by other diseases: acute pulmonary embolism, renal failure, dissecting aortic aneurysm, chronic HF, inflammatory diseases of the heart membranes (myocarditis, perimyocarditis), strenuous exercise, destruction of striated muscle tissue (rhabdomyolysis), the treatment of = underlying cancer using cardiotoxic chemotherapy, infiltrative disorders (e.g., amyloidosis), radiofrequency ablation therapy, defibrillator discharges, chest trauma, systemic inflammation (sepsis), shock, the exacerbation of chronic obstructive pulmonary disease and diabetic ketoacidosis [31]. In this regard, it is necessary to be extremely careful in interpreting the increased CTs serum levels and take into account data obtained by using other diagnostic methods (case history, physical examination, functional diagnostics) before conducting additional potentially dangerous invasive studies (coronary angiography), final diagnosis and therapy. The main reasons for the increase in CTs levels are shown in Figure 1.

With regard to the diagnostic contribution of CTs, it is necessary to note the importance of analytical characteristics of laboratory detection methods, the improvements of which led to changes in a number of ideas about the biochemistry, metabolism and the diagnostic significance of CTs. Modern methods for determining CTs are called high-sensitive methods, and their main advantage is a very low detection limit/limit of detection (LoD), which allows the early detection of CMCs damage. Thus, the average LoD of the hs-cTnI methods is about 2 ng/L [40], while that for hs-cTnT is 3–5 ng/L according to the incubation time of the method (i.e., STAT or Not STAT procedure) [41]. So, the earlier CMC damage is detected (increased levels of CTs), the earlier it can be diagnosed. However, for the diagnosis of MI, it is important to take into account the data of other diagnostic techniques (electrocardiography, echocardiography, etc.), indicating CMC ischemia. Unlike highly sensitive immunoassays, moderately sensitive troponin immunoassays have a higher LoD (average = 50–100 ng/L), so they can later detect CMC damage, which is a significant disadvantage.

In accordance with the recent international recommendations of experts of the International Federation of Clinical Chemistry (IFCC), highly sensitive troponin immunoassays should meet two main criteria: (A) CTs concentrations should exceed LoD in 50% (or more) of healthy people (for men and women) and (B) the coefficient of variation (CV) at the level of 99th percentile should not exceed 10% [42]. In addition to the main analytical characteristics (LoD, 99th percentile, CV, limit of blank (LoB)) of highly sensitive troponin immunoassays [43], the biological variations of CTs can play an important diagnostic role [44]. The main parameters of the biological variation of CTs are: individual biological variation (CVi), between-subject biological variation (CVg) and the index of individuality, which is calculated using a special formula: $Index\;of\;individuality=\frac{{\left(CV^{2}+CVi^{2}\right)}^{1/2}}{\mathrm{CVg}}$. If the index of individuality is ≥1.4, then it may be more clinically useful to interpret the result of a test using population-based reference values (99th percentile). If the index of individuality is <0.6, then this indicates a strong individuality of the circulating levels of CTs, and in this case the population values of the 99th percentile become unsuitable for clinical practice [44].

The following features of high-sensitive methods for determining CTs are of clinical significance: (1) early diagnosis of MI (within 1–2 h from the time the pain syndrome develops or from admission to the emergency department), whereas earlier, when using moderately sensitive methods for determining CTs, diagnosis took an average of 12–24 h [43]; (2) the role of gender, circadian and age factors, which, according to a number of studies, can affect the accuracy of diagnosis [34,35,36,37,38,39]; (3) the possibility to determine CTs in healthy people with risk of cardiovascular diseases or those with subclinical forms of cardiovascular diseases (for example, coronary heart disease, arterial hypertension, transient ischemic attacks, etc.), and increased levels of CTs in the cases of these diseases have a high predictive significance in terms of identifying an unfavorable prognosis [45,46,47,48]; and (4) the diagnostic significance of CTs concentrations in urine and oral fluid (saliva) in the case of cardiovascular disease [49,50,51,52,53,54,55,56]. Several recent clinical reports provide evidence of this new diagnostic capability [52,53,54]. That is, the authors found that the concentration of CTs (troponin I and troponin T) in the saliva of healthy individuals is significantly less than in patients with ischemic myocardial injury [52,53,54]. This new field of non-invasive diagnostics and monitoring of cardiovascular diseases has promising prospects due to a number of advantages associated with obtaining biomaterial.

One of the most common clinical forms of cardiovascular disease is arterial hypertension. This disease is also considered a risk factor for the development of acute cardiovascular and cerebrovascular diseases. Taking into account the high prevalence of arterial hypertension and its contribution in the development and progression of dangerous cardiovascular complications, the consideration of CMCs damage mechanisms and predictive significance of the main damage biomarkers (CTs) in case of this disease is of fundamental and practical importance, and therefore is especially noteworthy. The discussion of these aspects is the focus of this review.

### Materials and Methods

This is a descriptive review, which was prepared using the following databases: Embase, Pubmed/Medline and Web of Science. The following key words were used in the literature search: “myocardial injury” and “arterial hypertension” in combination with the terms “cardiac troponins” and “mechanisms of increase”.

## 2. Arterial Hypertension as a Significant Cause of Increased CTs: Mechanisms of CMCs Damage and CTs Increase

Arterial hypertension is one of the leading risk factors for the development of acute cardiovascular diseases, which result in the development of approximately half of the cases of acute coronary heart disease and acute ischemic strokes [57]. An additional negative factor of arterial hypertension is the perfidy of this disease, which is in the lack of symptoms in the early stages of its development and the gradual adaptation of patients to high blood pressure, so many cases of arterial hypertension remain underdiagnosed. As a result, the first clinically significant manifestations of arterial hypertension, which force patients to go to emergency departments, are acute cardiovascular complications, such as MI, transient ischemic attacks, strokes, etc. Thus, S. Caligiuri et al. detected high blood pressure in 50% of workers of a number of urban enterprises who had never previously complained of blood pressure and therefore did not take drugs to normalize it. In addition, systolic and diastolic pressures exceeded 180 and 120 mmHg in 2% of the tested persons—these values correspond to a hypertensive crisis and require emergency treatment [57].

According to a number of recent studies, arterial hypertension can be considered as a significant cause of increased CTs in both the blood serum [58,59,60] and urine of patients [61,62,63]; however, the pathophysiological mechanisms that cause an increase in CTs levels and CMCs damage have not been fully established. Taking into account the mechanisms of arterial hypertension development, the following key pathophysiological mechanisms can be identified that underlie CMCs damage and CTs release into the bloodstream: (A) myocardial hypertrophy due to increased load on the myocardium; (B) increased apoptosis due to the hyperactivity of the sympathoadrenal system or increased load on the myocardium; (C) damage to CMCs cell membranes, which leads to an increase in membrane permeability and CTs release; and (D) the effect of blood pressure on the glomerular filtration rate, which is important in the elimination of CTs from the bloodstream.

### 2.1. Myocardial Hypertrophy

High blood pressure causes an increase in preload on the myocardium, which causes its compensatory restructuring and the formation of hypertrophy, which is manifested by an increase in CMCs volume. With an increase in CMCs, the level of CTs released into the bloodstream increases as a result of the metabolism and renewal of CMCs [64,65,66]. This is evidenced by clinical studies that have revealed connection of myocardial hypertrophy with CTs levels. An additional indirect reasoning of this mechanism is also the research that revealed the gender characteristics of CTs levels [67,68]. At the same time, the main mechanism for the formation of gender characteristics, according to the authors, is the mass of the myocardium, which is higher in men than in women [68,69]. Similar features are also characteristic of the metabolism of striated skeletal muscles, which is manifested by gender differences in the levels of creatine phosphokinase and its MB isoform, skeletal troponins, creatinine, and other muscle tissue metabolites [70]. However, the most important proof of this mechanism functioning, i.e., the fact that more CTs molecules are released from hypertrophic CMCs are clinical studies that have revealed a correlation of CTs levels with myocardial hypertrophy in healthy individuals and patients with arterial hypertension [71,72].

### 2.2. Apoptosis of CMCs

The apoptosis of cardiac muscle tissue cells in case of arterial hypertension develops as a result of myocardium walls stretching, increased preload, and the hyperactivation of the sympathoadrenal system, which has been shown in a number of experimental and clinical studies [73,74,75,76]. Thus, W. Cheng et al., when studying the effect of myocardium stretch on the processes of CMCs apoptosis, noted that in CMCs the generation of reactive oxygen species (2.4 times) and Fas protein expression (21 times) increases from the stretch zone, which indicates a significant increase in apoptosis in response to myocardium stretch [73]. B. Weil et al., by increasing the preload on CMCs and increasing blood pressure by administering phenylephrine to laboratory pigs, also noted a significant activation of the programmed death of CMCs compared with animals that had placebo (31.3 ± 11.9 vs. 4.6 ± 3.0 CMCs in state of apoptosis/cm^2^; *p* < 0.01). In addition, a high degree of CMCs apoptosis in the experimental group was accompanied by a sharp increase in CT-I levels (856 ± 956 ng/L after 1 h and 1462 ± 1691 ng/L after 24 h) [74]. Finally, the hyperactivation of the sympathoadrenal system can be noted as another important factor that enhances CMCs apoptosis in arterial hypertension. Studies on cardiac myocytes in vitro showed that beta-agonists enhance CMCs apoptosis through the cAMP-dependent activation of voltage-operated calcium channels and overload of CMCs with calcium ions [75] and NF2-signaling pathway leading to the c-Jun activation of N-terminal kinases, increased levels of cytosolic cytochrome c and Bax expression [76].

### 2.3. Damage to CMCs Cell Membranes and Increased Permeability

**Figure 2:**
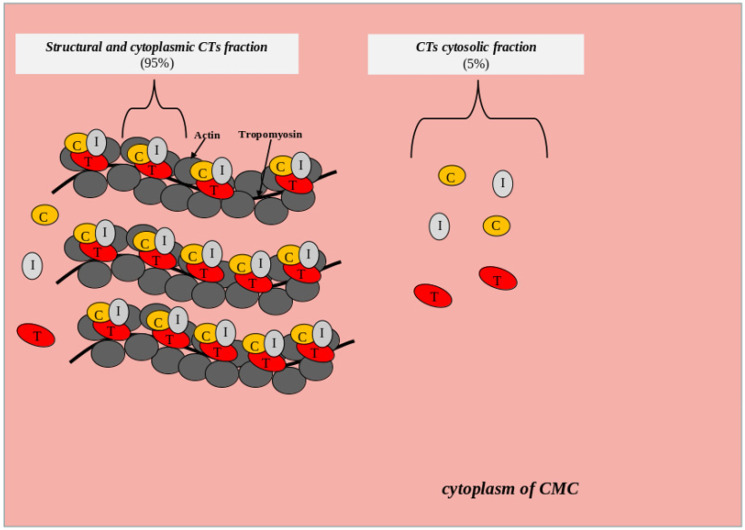
Scheme of localization of the structural and cytoplasmic CTs fraction. List of abbreviations: CTs—cardiac troponins; C—troponin C, T—troponin T, I—troponin I, CMC—cardiomyocytes.

The state of the cell membrane is one of the key factors affecting the degree of cytosolic protein molecules release into the bloodstream from the intracytoplasmic space. Many pathological conditions are accompanied by the release of cytoplasmic biomarkers into the bloodstream even before cell death (irreversible damage). For example, the release of liver enzymes (aspartate aminotransferase, alanine aminotransferase and gamma-glutamyl transferase) and muscle markers (creatine phosphokinase and its isoforms, skeletal troponin isoforms and myoglobin) in the early stages of inflammatory diseases of the hepatobiliary tract and skeletal myopathies, respectively, when there are no symptoms of necrosis yet [77,78]. CTs are localized both within the troponin system on thin filaments (structural or non-free CTs fraction) and freely (cytoplasmic or free CTs fraction). There is some evidence that approximately 95% of the total CTs content in CMCs is in the composition of the structural fraction and about 5% is in the composition of the cytoplasmic fraction (Figure 2).

A number of authors believe that CTs cytosolic fraction can exit the CMCs cytoplasm into the bloodstream with minor and reversible CMCs damage that occurs during physical activity [10] or under the influence of psycho-emotional stress [33]. The degree of increase in CTs concentrations in case of such damages is small, amounting to only 3–5 times the upper reference limit, which is due to the relatively small volume of freely localized CTs molecules in the cytoplasm. In addition, the CTs cytoplasmic fraction of CT is of significant importance in the formation of circadian rhythms in both healthy individuals and patients with cardiovascular diseases. The highest levels of CTs are observed in the morning, which is associated with the increased activity of sympathoadrenal system [79,80,81,82], hemostasis system [83,84,85,86] and the secretory activity of the thyroid gland [87,88,89]. According to M. Hessel et al., the CTs cytoplasmic fraction can be released with an increase in blood pressure and myocardial preload. The main mechanism responsible for this increase in CTs levels is CMCs transmembrane receptor proteins, in particular integrins. The stimulation of the latter is accompanied by the activation of enzymes (matrix metalloproteinases and calpain), which cause damage (increase in permeability) to the CMCs’ membrane and CTs release into the bloodstream [90]. In addition to increase in permeability of CMCs cell membrane, these enzymes can also promote CTs fragmentation into small fragments that can freely pass through the cell membrane [91,92]. This is proved by a number of clinical studies that have found small molecular forms of CTs in the blood serum of patients with MI [93] and renal insufficiency [94], as well as in healthy individuals after a marathon race [95]. Consequently, the mechanisms of degradation of CTs and their elimination may have important diagnostic significance. Arterial hypertension probably causes the degradation of CTs (due to the activation of enzymes (matrix metalloproteinases and calpain)); however, this needs further clarification.

### 2.4. Effect of Blood Pressure on Glomerular Filtration Rate and Elimination of CTs from the Bloodstream

The most important factors influencing the CTs levels in the blood serum are the mechanisms of their elimination from the bloodstream. At the same time, the urinary system is of significant importance in the elimination of CTs. A number of studies have reported a high prevalence of increased CTs levels in patients with urinary system diseases, in particular renal failure, which, therefore, should be considered as a significant and non-MI-related cause of increased CTs [19,96,97]. A correlation has been noted between the degree of renal failure, determined on the basis of glomerular filtration rate, and the degree of CTs increase in patients without symptoms of cardiovascular disease [19]. Factors that increase the glomerular filtration rate, in particular high blood pressure, on the contrary, increase the elimination of CTs from the bloodstream into the urine, as shown in the study by P. Pervan et al. [61]. This fact is of great practical importance, which is in the possibility of using urine as a biomaterial for monitoring the course of arterial hypertension and assessing its prognosis. Another factor affecting the glomerular filtration of proteins is the state of the renal (glomerular) filter. Damage to the latter in glomerulopathies often leads to increased protein excretion and, in particular, CTs.

One of the possible explanations for how CTs pass through the glomerular filter is the proteolytic cleavage of CTs under the influence of a number of intra- and extracellular proteinases. Thus, the size of a protein molecule is associated with the possibility of its passage through the small pores of the glomerular filter. Low molecular weight proteins, in contrast to high molecular weight proteins, are usually found in small amounts in primary urine, indicating a relationship between filtration and molecular size [95,96,97,98]. CTs under the influence of a number of intracellular and extracellular proteolytic enzymes are fragmented into several dozen fragments, the molecular weight of which is extremely small and probably allows them to be more actively eliminated from the bloodstream. CTs elimination is possible not only through the glomerular filter but also through other barriers, in particular, the hematosalivary, i.e., blood–brain into saliva and cerebrospinal fluid, which is confirmed by relevant studies [50,51,52,53,54,55,56,60,99,100]. However, the processes of the proteolytic cleavage of troponins inside cells and in blood serum have been studied very little. Although researchers have reported dozens of fragments of various molecular weights and sizes, all of the enzymes that are responsible for the cleavage of troponins and the formation of such a significant number of fragments are not known. There are extremely few studies that are focused on the specific mechanisms of proteolytic cleavage of CTs. One such study, conducted by Russian and Finnish researchers and led by I. Katruha [101], reported that the enzyme thrombin catalyzes the specific cleavage of CT-T into two fragments. It is noteworthy that under the conditions of high blood pressure in patients, the activation of this enzyme is observed [102] and, accordingly, the processes of the proteolytic cleavage of troponins into small fragments are intensified and the glomerular filtration rate is increased, which contributes to the elimination of formed small fragments of CTs through the glomerular filter from the bloodstream into the urine. The identification of all CTs that affect proteolytic cleavage is important for understanding this process and improving laboratory diagnostics, including the use of urine as a non-invasive biomaterial. Among the most valuable cardiac markers recommended for diagnosing MI and HF, the determination of CTs and natriuretic peptides (NUP) in urine has shown promising results. Recent studies by several research groups have shown the high diagnostic significance of CTs and NUP in non-invasively obtained body fluids in individuals with CHD [55,103], MI [52,53,54], DM [56], HF [104,105] and arterial hypertension [61]. Further research should be aimed at clarifying these promising diagnostic possibilities. 

**Figure 3:**
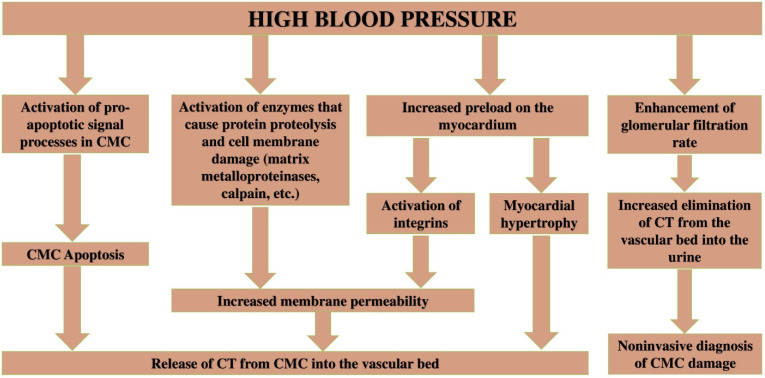
Pathophysiological mechanisms of CMCs damage and increased CTs levels in body fluids in case of arterial hypertension.

Summarizing the above, it is possible to propose the following pathophysiological mechanisms of CMCs damage and increased CTs levels in patients with arterial hypertension (Figure 3).

## 3. Predictive Significance of CTs in Patients with Arterial Hypertension

The mechanisms of CTs release into the bloodstream from CMCs are of great practical importance since high levels of CTs indicate damage to CMCs and an unfavorable prognosis for patients [59,60,61,106,107,108]. Data on the prevalence and predictive significance of CTs in the case of arterial hypertension based on the results of clinical trials are presented in Table 1.

## 4. Conclusions and Perspectives

Based on the results of literature analysis, arterial hypertension is considered as a very common pathological condition that causes damage to myocardial cells and is associated with a poor prognosis. An increase in CTs levels in body fluids (blood, oral fluid and urine), according to clinical studies, indicates damage to CMCs in the case of arterial hypertension. Damage to CMCs and an increase in CTs are caused by a number of pathophysiological mechanisms: (a) myocardial hypertrophy; (b) the death of CMCs as a result of apoptosis; (c) the activation of proteolytic enzymes inside CMCs, which leads to damage to cell membranes and an increase in membrane permeability; and (d) increased first stage of urinary formation (filtration), which is accompanied by an increase in the elimination of CTs from the blood into the urine.

A relatively new area of modern diagnostics is the study of the diagnostic/predictive significance of a number of biomarkers in non-invasively obtained body fluids. As the latter, oral fluid and urine are the most convenient, since their collection is atraumatic, non-invasive, painless and, in addition, does not require the participation of trained medical personnel in collecting this biomaterial. The development of testing sticks for biomarkers, in particular cardiomarkers, in the near future will allow rapid diagnostics at home to monitor the condition in cases of chronic cardiovascular diseases or the preliminary diagnosis of potentially dangerous acute forms of cardiovascular diseases before the arrival of an ambulance.

## Figures and Tables

**Table 1:** Clinical studies on the diagnostic significance of CTs in case of arterial hypertension.

Number of Patients, Diagnosis	Body Fluid under Study	Prevalence and Degree of CTs Increase in the Case of Arterial Hypertension, Predictive Significance	Source
Arterial hypertension, *n* = 306	Blood serum	Increased CT-T levels were observed in 47% of patients and were associated with left ventricular hypertrophy and poor long-term prognosis.	H. Uçar et al. [59]
Arterial hypertension, *n* = 467	Blood serum	Increased CT-I levels were observed in 15% of patients and were associated with the risk of chronic HF.	G. Acosta et al. [106]
Arterial hypertension, *n* = 171	Blood serum	Increased CT-I levels were observed in 32% of patients and were associated with the increased risk of major adverse cardiac events (MACE) and the risk of CHD.	D. Pattanshetty et al. [107]
Arterial hypertension, *n* = 929	Blood serum	Increased CT-I levels were observed in 31% of patients and were associated with the risk of MI and pulmonary edema.	M. Talha Ayub et al. [108]
Arterial hypertension, *n* = 20	Urine	CT-I levels in the urine of patients with hypertension were significantly higher than in patients with normal blood pressure. Reference intervals for CTs levels in the urine have not yet been established.	P. Pervan et al. [61]

List of abbreviations. CT—cardiac troponins; CHD—coronary heart disease; HF—heart failure; MI—myocardial infarction.

## Data Availability

Not applicable.
